# Spatiotemporal Patterns of White Matter Maturation after Pre-Adolescence: A Diffusion Kurtosis Imaging Study †

**DOI:** 10.3390/brainsci14050495

**Published:** 2024-05-13

**Authors:** Ezequiel Farrher, Farida Grinberg, Tamara Khechiashvili, Irene Neuner, Kerstin Konrad, N. Jon Shah

**Affiliations:** 1 Institute of Neuroscience and Medicine 4, INM-4, Forschungszentrum Jülich, 52425 Jülich, Germany; fgrinberg@live.de (F.G.); tamo.xechiashvili@gmail.com (T.K.); ineuner@ukaachen.de (I.N.); n.j.shah@fz-juelich.de (N.J.S.); 2 Department of Neurology, RWTH Aachen University, 52074 Aachen, Germany; 3 Department of Psychiatry, Psychotherapy and Psychosomatics, RWTH Aachen University, 52074 Aachen, Germany; 4 JARA—BRAIN—Translational Medicine, 52074 Aachen, Germany; kkonrad@ukaachen.de; 5 Child Neuropsychology Section, Department of Child and Adolescent Psychiatry and Psychotherapy, RWTH Aachen University, 52074 Aachen, Germany; 6 Institute of Neuroscience and Medicine 3, INM-3, Forschungszentrum Jülich, 52425 Jülich, Germany; 7 Institute of Neuroscience and Medicine 11, INM-11, JARA, Forschungszentrum Jülich, 52425 Jülich, Germany

**Keywords:** brain tissue microstructure, maturation, heterogeneity, heterochronicity, diffusion kurtosis imaging, diffusion MRI

## Abstract

Diffusion tensor imaging (DTI) enables the assessment of changes in brain tissue microstructure during maturation and ageing. In general, patterns of cerebral maturation and decline render non-monotonic lifespan trajectories of DTI metrics with age, and, importantly, the rate of microstructural changes is heterochronous for various white matter fibres. Recent studies have demonstrated that diffusion kurtosis imaging (DKI) metrics are more sensitive to microstructural changes during ageing compared to those of DTI. In a previous work, we demonstrated that the Cohen’s *d* of mean diffusional kurtosis (*d*_MK_) represents a useful biomarker for quantifying maturation heterochronicity. However, some inferences on the maturation grades of different fibre types, such as association, projection, and commissural, were of a preliminary nature due to the insufficient number of fibres considered. Hence, the purpose of this follow-up work was to further explore the heterochronicity of microstructural maturation between pre-adolescence and middle adulthood based on DTI and DKI metrics. Using the effect size of the between-group parametric changes and Cohen’s *d*, we observed that all commissural fibres achieved the highest level of maturity, followed by the majority of projection fibres, while the majority of association fibres were the least matured. We also demonstrated that *d*_MK_ strongly correlates with the maxima or minima of the lifespan curves of DTI metrics. Furthermore, our results provide substantial evidence for the existence of spatial gradients in the timing of white matter maturation. In conclusion, our data suggest that DKI provides useful biomarkers for the investigation of maturation spatial heterogeneity and heterochronicity.

## 1. Introduction

In recent decades, neuroimaging methods have provided valuable insights into structural changes in the developing brain in vivo, and numerous studies [1,2,3] have shown that morphometric parameters, such as whole-brain and regional brain volumes, and cortical thickness and surface area, reveal substantial changes throughout childhood and adolescence. In particular, a number of studies have shown that total brain volume tends to peak between 10 (girls) and 14 (boys) years of age and slightly decreases thereafter, remaining roughly stable until the mid-thirties [2,4,5]. Furthermore, cortical grey matter (GM) volume is known to be greatest during childhood and tends to follow an inverted U-shaped trajectory, with peak volumes reached by late childhood or early adolescence, depending on the region [5,6]. In contrast, WM volume continues to rise throughout childhood and adolescence and stabilises around the middle of the second decade [7,8]. Although not all studies report consistent results regarding the details of developmental trajectories [5], they show that WM volume increases for a longer time (into late midlife) in comparison to GM volume, suggesting protracted WM maturation [9,10]. 

The development of more advanced, quantitative MRI techniques [11,12,13,14], such as diffusion MRI, relaxometry, magnetisation transfer, and myelin water imaging, has enabled additional valuable information relating to microstructural changes in brain tissue and neuronal circuitry to be obtained. In particular, over the last decade, diffusion MRI studies have provided exciting new insights into maturation mechanisms, demonstrating ongoing brain remodelling through childhood and adolescence. However, more detailed information on the underlying biophysical mechanisms and related quantitative metrics is still required [15,16,17]. 

The sensitivity of water diffusion in the brain to microstructural tissue properties is the result of local geometrical barriers imposed by cellular membranes and organelles restricting diffusional propagation. Two key scalar indices provided by diffusion tensor imaging (DTI) [18,19] are the mean diffusivity (MD), a rotationally invariant measure of molecular propagation in space, and fractional anisotropy (FA), a measure of diffusion directionality linked to, among others, orientational axonal coherence and density. Using wide age ranges and large samples of subjects, numerous DTI studies [8,10,16,20,21,22,23,24,25,26,27] have provided evidence for a decrease in overall diffusivity measures and an increase in FA during childhood and adolescence, which continue into middle-age adulthood and are followed by an opposite trend in old age. In general, DTI metrics show patterns of cerebral maturation and decline, rendering non-monotonic lifespan trajectories with age. These are mainly characterised by three stages: (a) the fastest changes, i.e., decreases in diffusivity and increases in FA values, are observed during the first two years of life, followed by strongly decelerating progression of the same trend throughout childhood and adolescence; (b) relative stability in early-to-middle-adulthood; and (c) a subsequent acceleration of the opposite trend, i.e., increases in diffusivity and decreases in FA values, in senescence. 

Among the DTI invariants investigated in age-related studies, the focus has frequently been on FA [21,28,29] as a putative marker that reflects various underlying microstructural features, such as axonal myelination, the orientational coherence of fibre bundles, packing density, etc. In particular, myelination plays a crucial role in the transmission of electrical signals and is linked to the efficiency of neuronal communication. Generally, WM maturation is associated with the development of cognitive functions during childhood [30,31]. Accordingly, correlations have been reported between FA and cognitive performance or deficits, such as in processing speed, memory, and reading ability [32,33,34,35,36,37,38,39]. 

Developmental trajectories of DTI metrics have been shown to be different for various anatomic regions and have been associated with their role in the maturation of executive functions and changes in brain circuitry [20,40]. Furthermore, they can be modelled as both linear and non-linear (quadratic, Poisson, and exponential) functions of age [10,21,22,41]. In particular, FA tends to follow an inverted U-shaped trajectory for several major tracts, such as the corona radiata and cingulum, reaching individual peak maxima in early-to-middle adulthood, most typically during the second and third decades [21,22,41]. Conversely, diffusivity measures in these fibres reveal U-shaped curves, with the minima found in the same decades but not necessarily at the same age as the peak for FA. Some other WM fibres, such as the fornix, centrum semiovale, or superior fronto-occipital fasciculus show linear decreases in FA or remain flat with age (range ca. 5–30 years). The observed magnitude of changes attained by different fibres during different periods were consistent with the tendency for late maturation of association (AF) in comparison to commissural (CF) and projection (PF) fibres [41].

More generally, the current understanding of developmental trajectories entails a complex spatiotemporal dynamical pattern [42]. Cortical GM development primarily advances in a posterior-to-anterior (P-A) direction, with myelination in WM proceeding concurrently with the development of overlaying GM and the progression of cognitive functions [43,44,45,46,47,48,49,50,51,52,53]. These works, including both anatomic MRI and DTI studies, have shown that posterior–inferior areas tend to underlie the initial emergence of basic sensory and motor functions, whereas anterior–superior areas develop later and support higher-order executive functions and multi-modal integration. In recent studies, Colby et al. [44] (32 subjects, 3–28 years old) and Krogsrud et al. [54] (159 children, 4–11 years old) investigated developmental timings in WM maturation as captured by DTI metrics. These studies have provided further evidence for an orderly maturation progression along P-A, central-to-peripheral (C-P), and inferior-to-superior (I-S) spatial directions. 

For nearly two decades, diffusion kurtosis imaging (DKI) [55,56], a higher-order extension of DTI, has gained increasing attention as a method for providing sensitive biomarkers of development [57,58,59,60], ageing [61,62,63,64,65,66], and various brain pathologies, such as stroke [67,68,69,70,71,72], neurodegenerative diseases [73,74,75], childhood epilepsy [76,77,78], and attention-deficit/hyperactivity [59,79]. In relation to developmental processes, strong increases in both FA and mean kurtosis (MK) have been reported for multiple WM regions during the first two years of life, with a subsequent slowdown toward a plateau-like behaviour [58]. The authors associated the increase in FA with a dominating myelination and axonal packing process. Interestingly, in contrast with FA, MK continued to rise beyond the 2-year mark and converged to a plateau at a later age, thus reflecting ongoing remodelling of the tissue microstructural environment. A further increase in MK beyond childhood/adolescence, as opposed to an ageing-related decrease [61,64] after the second decade of life, has only been reported in a relatively small number of works [57,59,62,66], emphasising the need for further studies. In particular, Falangola et al. [62] showed that MK in the prefrontal brain region was higher in young adults (26–47 years old) than in adolescents (12–17 years old). An increase in MK between the ages of 12 to 18 years, which was also limited to the prefrontal cortex, was reported by Helpern et al. [59] for a small group of subjects (*n* = 13). Age trajectories of DT and kurtosis tensor (KT) metrics in 21 WM and GM anatomic regions were investigated by Das et al. [66] in a work that had ageing processes as the primary focus.

In our previous work [57], we performed a comparative analysis of KT metrics in a group of children and a group of middle-aged adults for 20 anatomic WM regions provided by the Johns Hopkins University (JHU) Atlas—available in the FSL toolkit (http://fsl.fmrib.ox.ac.uk/fsl/fslwiki/Atlases, accessed on 7 May 2024). We demonstrated that KT metrics exhibited significant between-group differences and that they were of substantially larger magnitude than those observed with DT metrics. Quantified in terms of the Cohen’s *d* of MK (*d*_MK_), these differences showed strong heterochronicity maturation of the different fibres and, especially, the different fibre types. The largest *d*_MK_ values were observed for all (seven) AFs studied, such as the cingulum and superior longitudinal fasciculus, which is in agreement with their anticipated protracted maturation. The smallest *d*_MK_ values were observed in two of the CFs studied, such as forceps major and forceps minor, indicating more advanced maturation. Two of the PFs studied exhibited intermediate *d*_MK_. Thus, our results suggest that *d*_MK_ is highly sensitive to subtle maturation between pre-adolescence and adulthood and further indicate its ability to access the maturation grades of different fibre types. Driven by the interest in this phenomenon, this present paper extends the investigation of the differences between DT/DK metrics in children and adults to 27 WM regions provided by the JHU Atlas “ICBM-DTI-81” available in FSL. Furthermore, the spatiotemporal microstructural reorganisation of brain tissue through late childhood into adulthood, as revealed by DKI metrics, is explored and the potential of *d*_MK_ as a biomarker of its maturation grade is tested. Additionally, the potential of KT metrics to gain insights into major maturation gradients along the P-A, C-P, or I-S spatial directions is investigated. 

## 2. Materials and Methods

### 2.1. Subjects

Two groups of healthy volunteers, 20 children (range, 9–12 years, mean age, 10.3) and 21 adults (range, 38–64 years, mean age, 54.3) underwent diffusion MRI. 

### 2.2. Experiments

In vivo diffusion MRI experiments were performed with a whole-body 3T Siemens MAGNETOM Tim-Trio scanner (Siemens Medical Systems, Erlangen, Germany). The body coil was used for RF transmit, and a 12-element phased-array coil provided by the manufacturer was used for signal receive. The gradient system allowed for a maximal gradient strength of 40 mT/m and a slew rate of 200 T/m/s. As described in Grinberg et al. [57], diffusion-weighted images (DWIs) were acquired using a double spin-echo echo-planar imaging pulse sequence with the following protocol parameters: diffusion-encoding gradient directions = 30; 3 diffusion-weighting factors (*b*-values) = 0, 1, and 2.8 ms/µm^2^; TR = 10,900 ms, TE = 112 ms, pixel bandwidth = 1628 Hz/px, number of repetitions = 3, total acquisition time = 33 min, matrix size = 128 × 128 × 72; and voxel size = 1.9 × 1.9 × 1.9 mm^3^.

### 2.3. Data Processing and Statistical Analysis

DT scalar invariants (MD, axial (AD), and radial (RD) diffusivities and FA) and specific KT measures (MK, axial (AK), and radial (RK) kurtoses and kurtosis anisotropy (KA)) were determined on a voxel-by-voxel basis, within the framework of the DKI analysis [56,80]. The post-processing steps are described in detail elsewhere [57]. In brief, DWIs were corrected for eddy-current distortions and head motion using the FDT toolkit available in FSL [81]; signal bias due to background noise [82] was reduced following the method in Refs. [83,84]; DT/KT metrics were evaluated with the help of the ExploreDTI toolkit [85]. Individual FA maps were aligned to the FA template in the JHU space using the linear (firstly) and non-linear (secondly) registration approaches available in FSL. The estimated affine transformations and warp fields were then applied to the non-FA images. 

For the atlas-based analysis, the DT/KT metrics within each of the WM tracts provided by the JHU Atlas “ICBM-DTI-81” were averaged with no distinction between the left and right sides. In total, 27 tracts were considered and are listed in Table 1. Among the fibres studied in this (ICBM-DTI-81 WM labels atlas) and previous [57] (JHU WM tractography atlas) works, only five fibres (CST, Cg, Ch, SLF, and UF) were the same in both atlases. The values of the DT/KT parameters for these fibres in both works were in good agreement, and only small differences (~5–10%) in absolute values, easily attributable to the differences in the corresponding template ROIs, were observed. 

The following metrics were evaluated for each anatomically defined structure and for each DT/KT parameter:

(a) Relative changes (Δ*A*) in percentage between the group mean parameter values (*Ā*) (i.e., averaged over all voxels for a given anatomy and a given subject and then averaged over the whole group of subjects) according to Δ*A* = 100 × (*Ā*_adult_ − *Ā*_child_)/*Ā*_child_, where *A* indicates one of the DT/KT parameters;
(b) *p*-values of the between-group two-sided Student’s *t*-test analysis. In the following, we shall refer to statistical between-group differences as significant if *p* ≤ 0.00185 (after Bonferroni correction for multiple comparisons, *n* = 27);
(c) Between-group, age-related effect sizes using Cohen’s *d* [86] for each anatomically defined structure. The subscript of Cohen’s *d* indicates the parameter for which it was evaluated, i.e., *d*_MK_ is Cohen’s d for MK, *d*_FA_ is Cohen’s *d* for FA, and so forth.

In order to produce correlation plots between *d*_MK_ and age of extrema in the life trajectories of FA and MD, we used the values of age of peak for FA and age of minimum for MD from Table 2 in Lebel et al.’s study [22]. 

The profiles of between-group, relative differences in the directions right-to-left (R-L), P-A, and I-S were constructed using the following procedure: all individual FA maps were linearly and non-linearly registered to the MNI152 FA standard space available in FSL (1 × 1 × 1 mm^3^) (http://fsl.fmrib.ox.ac.uk/fsl/fslwiki/Atlases, accessed on 7 May 2024). The corresponding affine transformations and warp fields were then applied to the remaining DT/KT parameter maps. For each DT/KT parameter, the maps of the group-average DT/KT parameters for children and adults, along with the corresponding maps of the between-group relative differences in percentage, were evaluated on a voxel-by-voxel basis, yielding the group average (${\bar{A}}_{v}$, where “v” denotes “voxel-by-voxel”) and the group-difference maps ($\Delta {\bar{A}}_{v}$). These parameters were then projected onto the group-wise FA skeleton, which was produced with the help of TBSS (tract-based spatial statistics), available in FSL [87]. In order to generate profiles along each of the three axes (R-L, P-A, and I-S), the group-difference maps, $\Delta {\bar{A}}_{v}$, were averaged over all voxels in the skeleton within each individual plane perpendicular to a given direction (R-L, A-P, or I-S), yielding one average value per plane ($\langle \Delta {\bar{A}}_{v}\rangle$, where $\langle \ldots \rangle$ indicates averaging over a given plane). These values were then plotted as a function of the plane position. To simplify visualisation, we subtracted the mean values of $\langle \Delta {\bar{A}}_{v}\rangle$ averaged over the entire profile from the ordinates so that all profiles appear “centred” around zero. The colour of individual data points indicates whether the *t*-test of between-group differences performed for the values of ${\bar{A}}_{v}$ (considering only skeleton voxels of the corresponding plane) was significant (red) or non-significant (blue) with (threshold) α values set to 0.05. Finally, the evaluated parameter gradient profiles were fitted using polynomials of first (linear) and second (quadratic) order. To investigate the C-P direction (i.e., from the centre to the left or right periphery), the data sets from the R-L direction were subdivided into left and right halves of the whole profile and fitted separately. Additionally, the whole R-L profile was fitted using a second-order polynomial. The goodness-of-fit was assessed using the values of coefficient of determination (R^2^), F-statistics (F-stat), and *p*-values provided by the linear regression model algorithm available in Matlab (for more detail, see the function fitlm under www.mathworks.com/help/stats/fitlm.html, accessed on 7 May 2024).

## 3. Results

### 3.1. Whole-Brain WM Histograms and Atlas-Based Analysis of WM Tracts

**Figure 1:**
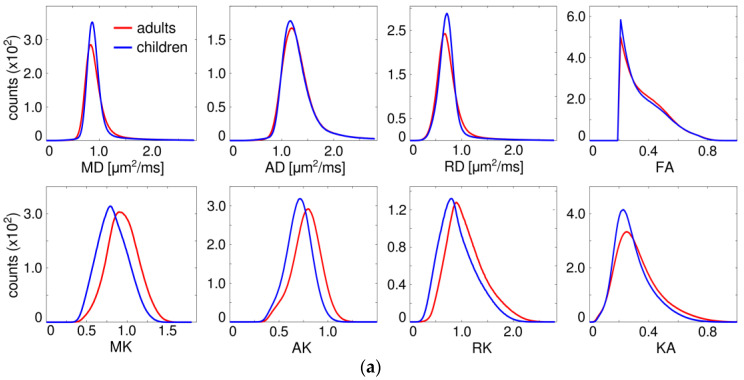

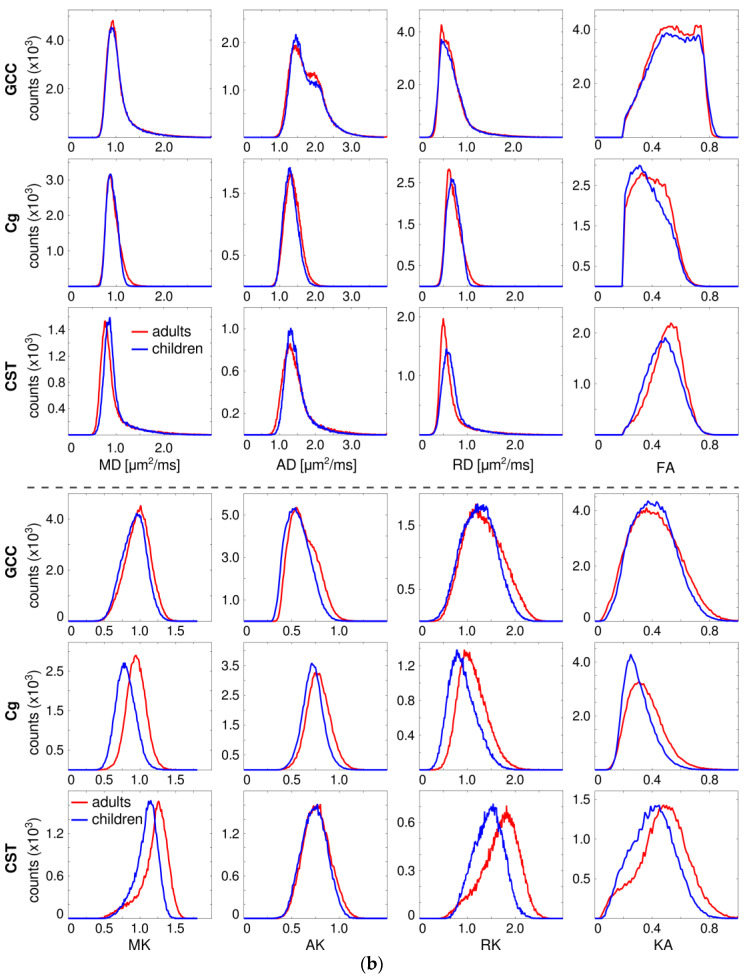
(**a**) Whole-brain histograms of the DT/KT metrics averaged within the groups of children and adults; (**b**) average histograms of the DT/KT metrics for three selected fibres: the Cg (AF), CST (PF), and GCC (CF).

Figure 1a shows the histograms of the whole-brain WM (FA > 0.2) averaged over the group of adults and the group of children. The histograms of the DT metrics appear largely overlapped, whereas the histograms of the kurtosis indices demonstrate clear between-group shifts. This effect is even more pronounced in the histograms of individual fibres, as shown for three selected tracts representative of each of the AF (Cg), PF (CST), and CF (GCC) fibre types (Figure 1b).

**Figure 2:**
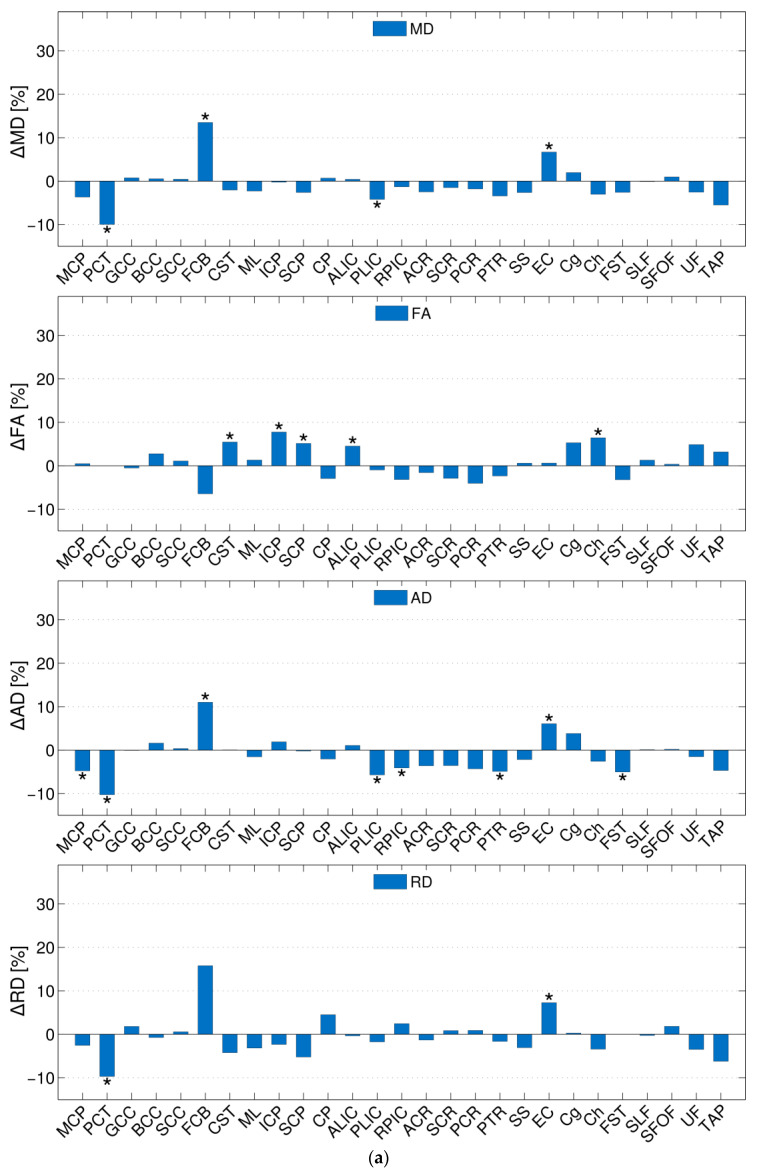

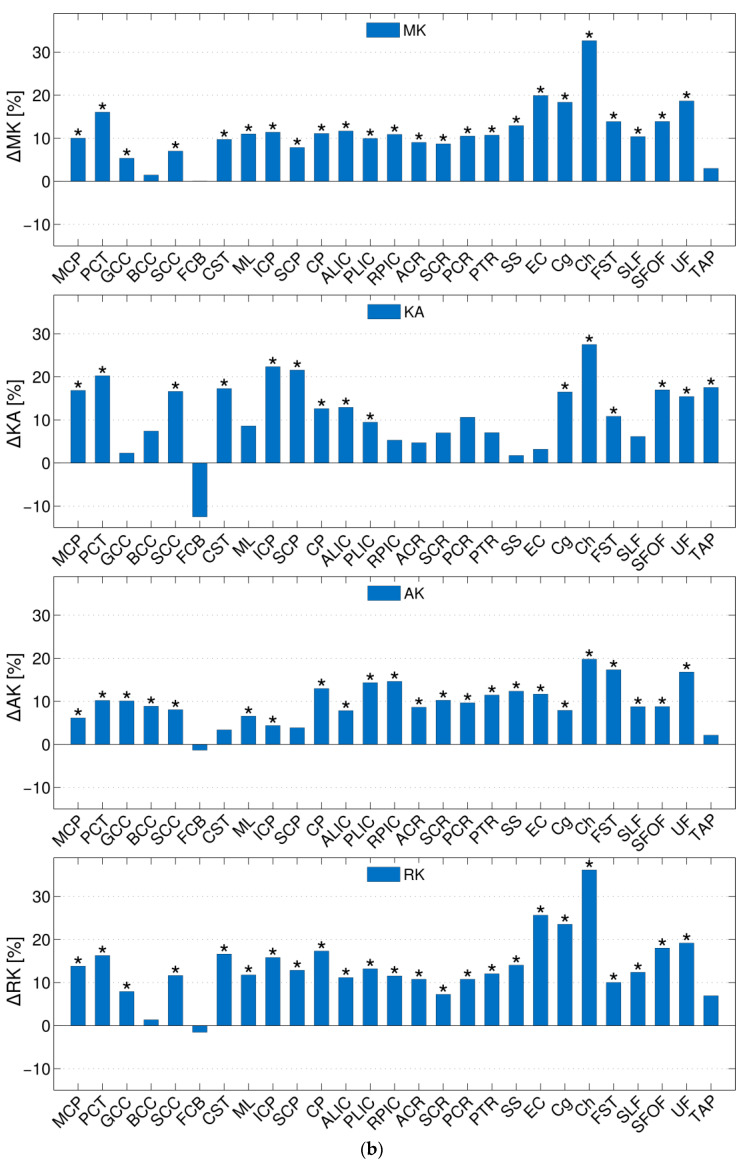
(**a**) Relative changes in the DT parameters in adults and children for different fibres; (**b**) relative changes in the KT parameters in adults and children for different fibres. Significant between-group differences are indicated by asterisks based on the two-sided Student’s *t*-test analysis (*p* ≤ 0.00185, Bonferroni corrected).

The group mean values of the DT/KT parameters and the standard deviations for various WM fibres are presented in Supplementary Table S1 (DT) and Table S2 (KT). Relative between-group differences in the parameter means are shown in Table 2 (DT) and Table 3 (DK). As an overview, the relative differences in percentage are visualised as bar plots in Figure 2a (DT) and Figure 2b (KT), where asterisks indicate significant (*p* ≤ 0.00185) between-group differences. For each individual fibre, the between-group differences were considerably larger for KT than for DT metrics. The magnitudes of the observed absolute differences between the group means in any DT metrics were relatively small on average across the fibres: approximately 2.9% for FA and MD and 3.2% for AD and RD. The largest observed changes in the FCB were equal to 13.5% in MD, 11.0% in AD, and 15.8% in RD, whereas in the ICP, the largest change was equal to 7.8% in FA. None of the fibres exhibited significant differences in all DT parameters, but several fibres showed statistically significant differences in some of the metrics: CST (FA), ICP (FA), SCP (FA), ALIC (FA), Ch (FA), PCT (MD, AD, RD), FCB (MD, AD), PLIC (MD, AD), EC (MD), MCP (AD), RPIC (AD), PTR (AD), EC (AD), and FST (AD). In contrast, the magnitudes of the absolute changes in the KT parameters were rather large; that is to say, on average, across the fibres, they were equal to ca. 11.3%, 9.6%, 13.7%, and 12.3% for MK, AK, RK and KA, respectively. The largest differences between the group means were observed for the same fibre, i.e., the Ch, in all KT metrics, and were as large as 32.7% for MK, 19.8% for AK, 36.2% for RK, and 27.5% for MK, AK, RK, and KA. The between-group mean KT parameter differences were significant for most of the fibres, with *p*-values as low as ~10^−15^ for MK in Ch, for example. Out of all of the fibres examined, only a few fibres, namely, the BCC (MK, AK, and KA), FCB (all KT metrics), TAP (MK, AK, and RK), CST (AK), and SCP (AK), did not show statistically significant differences in one or more KT metric. Additionally, ten other fibres (GCC, ML, RPIC, ACR, SCR, PSR, PTR, SS, EC, and SLF) did not show significant differences in only the KA parameter.

The Cohen’s *d* values were considerably larger for the KT parameters than for the DT parameters, as clearly seen from a comparison of Table 2 and Table 3. According to the conventional rule of thumb for the classification of effect sizes [88], on average, the absolute Cohen’s *d* values across the fibres were “large” ($\left|d\right|\geq \;$0.8) for all four KT parameters and were equal to 2.17 (MK), 2.02 (AK), 1.90 (RK), and 1.28 (KA); whereas the corresponding DT parameter values were equal to 0.55 (MD), 0.75 (AD), 0.43 (RD), and 0.58 (FA). Regarding the individual tracts, the Cohen’s *d* values were “large” in 24 (MK), 24 (AK), 24 (RK), and 17 (KA) fibres or “medium” (0.5 ≤ $\left|d\right|$ < 0.8) in 0 (MK), 1 (AK), 0 (RK), and 6 (KA) fibres, in comparison to 7 (MD), 5 (AD), 4 (RD), and 9 (FA) fibres with a “large” *d* or 6 (MD), 5 (AD), 7 (RD), and 7 (FA) fibres with a “medium” *d*. Moreover, a closer differentiation shows that the absolute Cohen’s *d* values were > 2.0 in 18 (MK), 15 (AK), 11 (RK), and 6 (KA) fibres (that is, a “huge” effect size according to a more expanded classification [89]), whereas they did not exceed 2.0 in any fibre for any DT metric. 

Pearson’s correlations between Cohen’s *d* values for various DT and KT pairs of parameters are shown in Table 4. In particular, *d*_MD_ was strongly correlated (*r* > 0.5) with *d*_AD_ (*r* = 0.89) and *d*_RD_ (*r* = 0.85) but not with *d*_FA_ or Cohen’s *d* of any KT metrics. Moreover, *d*_MK_ was strongly or moderately correlated with *d*_AK_ (*r* = 0.56), *d*_RK_ (*r* = 0.88) and *d*_KA_ but not with the Cohen’s *d* of any DT parameter. Additionally, *d*_RD_ was strongly negatively correlated with *d*_FA_ (*r* = 0.50) and *d*_KA_ (*r* = 0.60). Finally, *d*_RK_ was strongly correlated with *d*_KA_ (*r* = 0.63) and moderately correlated with *d*_FA_.

### 3.2. Assessment of Maturation Based on d_MK_

**Figure 3:**
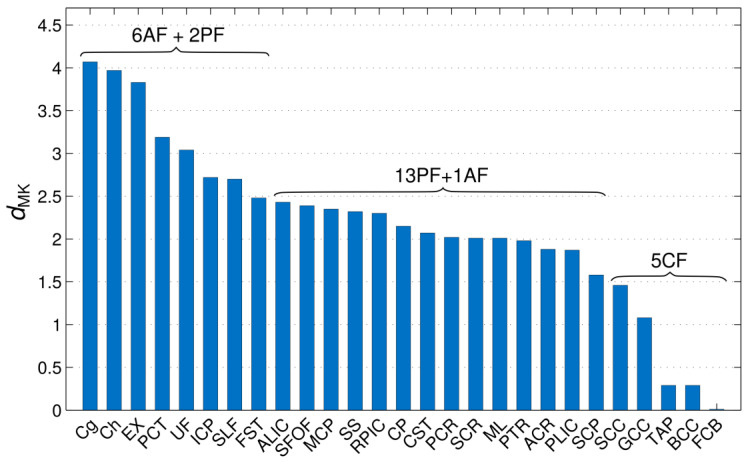
The values of *d*_MK_ for various fibres used to characterise the maturity of these fibres with respect to their microstructural changes between childhood and adulthood. The values are shown in descending order. The fibres with the highest *d*_MK_ are assumed to exhibit the most protracted maturation. AF, PF, and CF denote association, projection, and commissural fibres, respectively.

Figure 3 shows the bar plot of *d*_MK_ values for all investigated fibres. The range of the observed values varies from ~0 for the FCB to ~4 for the Cg and Ch. The five largest *d*_MK_ values were observed in the Cg, Ch, EC, PCT, and UF, and the five smallest ones were observed in the FCB, BCC, tapetum, GCC, and SCC. That is, all five fibres with the smallest *d*_MK_ are CFs, whereas four out of five with the largest *d*_MK_ are AFs. Taken separately, the mean *d*_MK_ values across AFs, PFs, and CFc were equal to 3.10 ± 0.75, 2.18 ± 0.40, and 0.62 ± 0.61, respectively. The quartile analysis shows that the first quartile (*d*_MK_ = 1.87) includes all of the CFs studied plus two PFs (SCP and PLIC), the range between the first quartile and the median (*d*_MK_ = 2.15) includes nine PFs, the range from the median to the third quartile (*d*_MK_ = 2.58) includes three more PFs and three AFs, and the range between the third and fourth quartiles includes six AFs and two PFs. Thus, there is a clear tendency for *d*_MK_ to enable the different fibres to be partially sorted in such a way that the AFs predominantly occupy the top (largest *d*_MK_, the most protracted maturation), the CFs predominantly occupy the bottom (smallest *d*_MK_, the most complete maturation), and the PFs predominantly occupy the middle of the descending order. 

**Figure 4:**
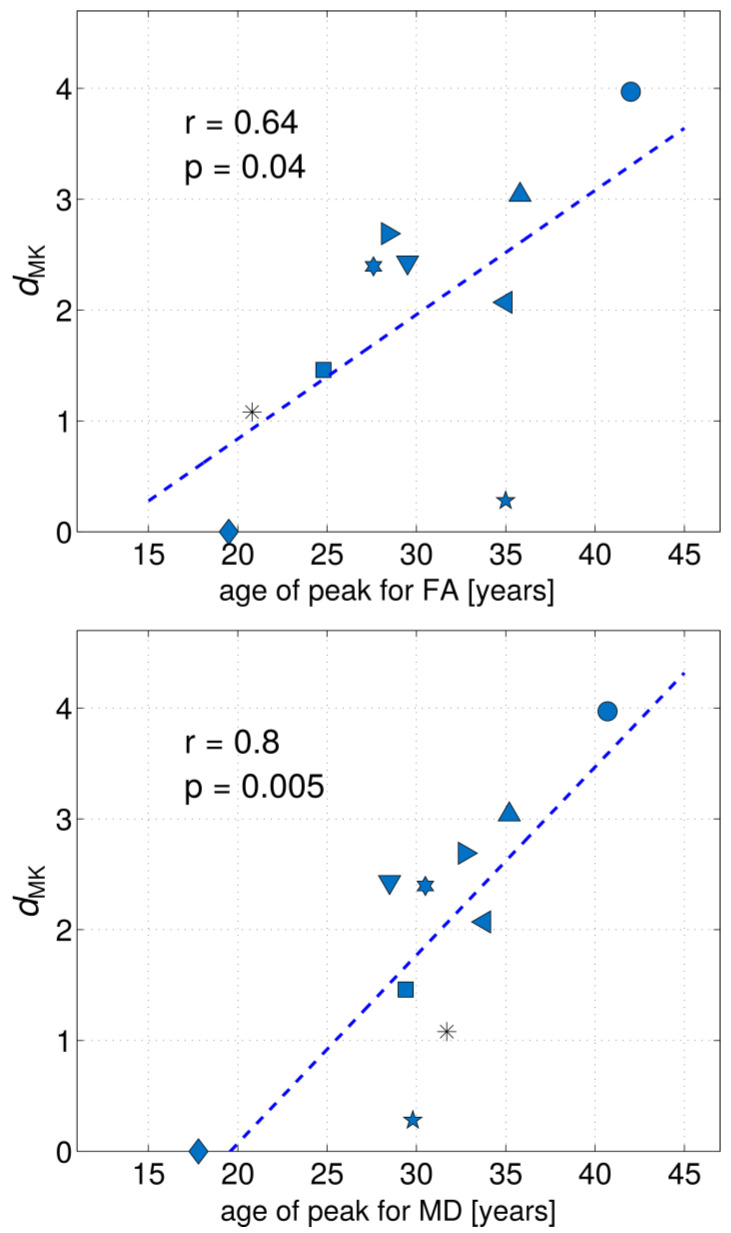
Scatter plots of *d*_MK_ versus age of peak for FA and age of minimum for MD for 10 investigated fibres. Herein, we used the data published by Lebel et al. [22] for the age of peak and age of minimum. Pearson’s correlation coefficients, *r*, are indicated on the plots and provide evidence of strong correlations between *d*_MK_ and the age at which FA and MD reach their extreme values. ▼, ALIC; ◀, CST; *, GCC; ■, SCC; ★, BCC; ◆, FCB; ●, Cg; ✶, SFOF; ▶, SLF; ▲, UF.

Figure 4 shows the scatter plots of *d*_MK_ versus age of peak for FA and age of minimum for MD for the ten investigated fibres. Herein, we used the values published by Lebel et al. [22] in Table 2 for age of peak and age of minimum. The respective Pearson’s correlation coefficients, *r*, were equal to 0.64 and 0.8, providing evidence of a strong correlation between *d*_MK_ and the age at which FA and MD reach their extreme values. 

### 3.3. Patterns of Developmental Change along C-P, P-A, and I-S Directions

**Figure 5:**
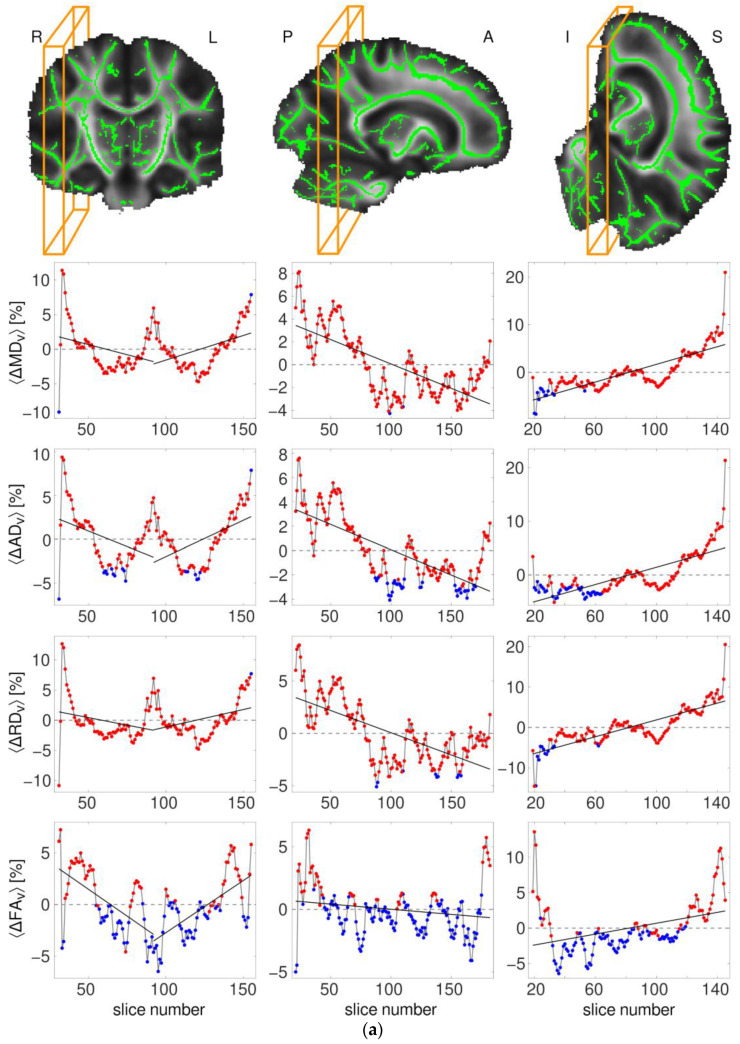

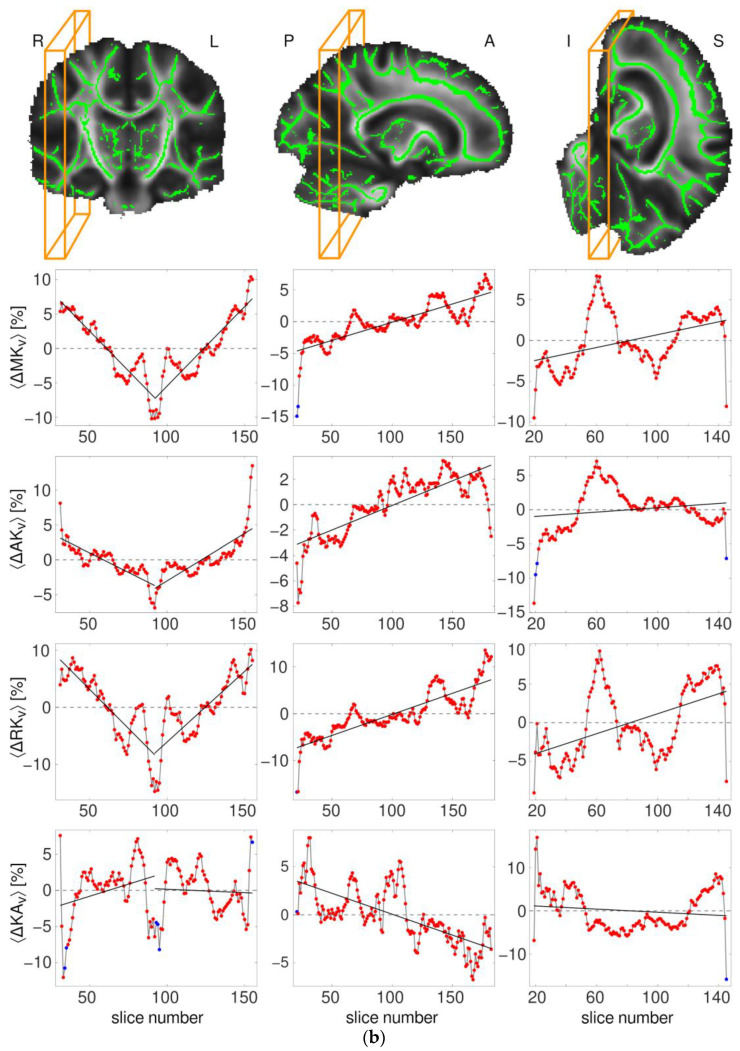
(**a**) Profiles of $\langle \Delta {\bar{A}}_{v}\rangle$, where *A* denotes one of the DT parameters indicated at the vertical axes; (**b**) profiles of $\langle \Delta {\bar{A}}_{v}\rangle$, where *A* denotes one of the KT parameters indicated at the vertical axes. All profiles appear “centred” around zero since the mean values of $\langle \Delta {\bar{A}}_{v}\rangle$ averaged over the entire profile were subtracted from the ordinates (to simplify visualisation). The colour of individual data points indicates whether the *t*-test of between-group differences performed for the values of ${\bar{A}}_{v}$ (considering only skeleton voxels of the corresponding plane) was significant (red) or non-significant (blue) with (threshold) α values set to 0.05.

Plane-by-plane profiles of percentual between-group differences in the mean DT and KT metrics along the indicated directions are shown in Figure 5a and Figure 5b, respectively. It can be seen that the profiles of the KT metrics are predominantly composed of red points (significant between-group differences, see Section 2.3), whereas those of DT metrics contain a considerable number of blue points (non-significant differences). Visual inspection exposes certain characteristic patterns, which are most pronounced in the C-P and P-A directions. The first striking difference can be seen between the C-P (first column) and the two other directions (second and third columns). In the C-P direction, KT metrics (except KA) show rather symmetric V-like patterns descending from the peripheral towards the central regions. The DT metrics also descend roughly from the periphery toward the centre but exhibit an additional symmetric upward change in the middle (rendering a more W-like pattern). Due to this characteristic pattern, the left and right halves of the profiles in the C-P direction were fitted and considered separately. In the P-A and I-S directions, the observed KT profiles are ascending, with the exception of those of KA. DT metrics provide a mixed picture, descending in P-A and ascending in I-S directions. Quantitative fit characteristics (R^2^, F-stat, and *p*-values) for linear and quadratic functions are shown in Table 5. F-statistics show that both linear and quadratic functions were significant for all parameters in all directions, with the exception of RD in the R-L (right) direction and KA in the R-L (left) and I-S directions. 

## 4. Discussion

Characterising WM microstructure at different stages of maturation can deepen our understanding of how the development of cognitive functions, behaviour, and emotions are supported by an underlying anatomic substrate [49,90,91]. It may also help elucidate the heterogeneity/heterochronicity of regional maturation in relation to brain functionality. This work substantially advances our preliminary study [57] and compares the potential of DT and KT parameters to assess the microstructural changes in WM between (pre-adolescent) childhood and middle adulthood. It further demonstrates the use of KT metrics as sensitive biomarkers of maturation beyond pre-adolescence. 

Generally, our findings are in agreement with previous DTI studies [7,10,21,92], indicating protracted maturation of widespread WM structures into the third/fourth decades and providing evidence of considerable regional heterochronicity. However, our results also demonstrate that KT indices are much more sensitive indicators of ongoing subtle microstructural development in comparison with DT parameters, thereby significantly enriching the palette of maturation-sensitive MRI tools. In the following, we shall discuss some noteworthy observations of heterochronicity, the correlation with age of peak, the maturation ranking of various fibres/types, and potential neurobiological mechanisms in the context of our experimental findings. 

### 4.1. DT Metrics

The range of percentual changes (between 0 and ~10–15%) observed in our work for various DT metrics and fibres generally agreed with typically reported findings [8,10,16,21,22,24,43]. However, a direct quantitative comparison is difficult because of the considerable differences between the investigated age ranges, selection of fibres, and applied methodical approaches used in the different works. In particular, the average percentual changes across all studied fibres (~3%) for both FA and MD in our work were somewhat smaller than the average 8% (FA) and 10% (MD) changes reported by Lebel et al. [22]. However, this can be easily attributed to differences both in the age ranges and in the selected anatomic regions (of which only a part was the same in both works). For example, the age range studied by Lebel et al. [22] included younger children, starting at five years old. Conversely, our group of children (9–11 years old) misses a portion of more rapid microstructural changes seen in earlier childhood and is closer to the transition age, where these changes become slower and continue to evolve more gradually, with a linear or non-linear dependence on age [10,21,22]. Furthermore, the reference data is evaluated differently, i.e., in our work, it is represented by average data for the range of 38 to 64 years old (mean age, 54.3 years), whereas Lebel et al. [22] consider the data until the peak of FA or the minimum of MD, which is usually reached between the second and third decades of life. In this context, one should consider that the patterns of lifespan trajectories [10,21,22,92] in fibres with non-linear age-dependence suggest that FA/MD changes in the range between circa ten years old and the age of extrema may be partially compensated by slow, opposite changes during the period of relative middle age stability. Moreover, not all WM regions exhibit such non-linear age-dependence in the relevant age interval but rather reveal a more linear behaviour [10,21,66]. Taken together, these factors may easily explain some differences between our findings and those reported in the aforementioned works. 

### 4.2. KT Metrics

All KT parameters (MK, RK, AK, and KA) significantly increased from childhood to adulthood in the majority of the investigated tracts and demonstrated an enormously large magnitude of changes, such as average (across all fibres) values of *d*_MK_, *d*_AK_, and *d*_RK_ > 2.0 and *d*_KA_ > 1.0, in comparison to moderate, < 0.8, average Cohen’s *d* of DT metrics. It is also worth mentioning that the *t*-test analysis provided much lower *p*-values for the between-group differences in KT compared to the DT metrics. This finding is important when considering the minimum group size required for statistical comparisons [93] during study planning. In particular, for a sample of 41 subjects, as in our work, the minimum detectable effect size for the two-tailed test is equal to 0.9 (provided the statistical power is set to 0.8 and the probability of making a Type I error is set to 0.05; see Table A1.1 in Ref. [88]). In our study, the Cohen’s *d* values ≥ 0.9 were relevant for the majority of the 27 fibres investigated and for all four KT metrics. Only three fibres (tapetum, BCC, and FCB) showed *d*_MK_ and *d*_RK_ < 0.9, three fibres (CST, tapetum, and FSB) showed *d*_AK_ < 0.9, and ten fibres showed *d*_KA_ < 0.9. In contrast, the Cohen’s *d* values of DT metrics were <0.9 in the majority of the fibres (i.e., in 21 fibres for *d*_FA_ and *d*_MD_, in 17 fibres for *d*_AD_, and in 24 fibres for *d*_RD_). Thus, a much larger sample size is required for statistically powerful studies based on DT parameters in the studied age range, increasing the overall expenditure and effort. As an example, very large sample sizes, such as 831 [21] or 3513 [94] subjects, have been used in some large-scale, age-related DTI studies. 

### 4.3. Maturation “Ranking” Based on d_MK_

As first suggested in our previous work [57], *d*_MK_ can be used for the assessment of fibre maturation: large values are indicative of more protracted development, whereas low values indicate more complete maturation. The observed *d*_MK_ values clearly confirmed essential heterochronicity in the maturation of various fibres. Moreover, they allowed for the partial sorting of maturation levels reached by the age of pre-adolescence in different fibre groups, such as CFs, PFs, and AFs. The differentiation grade of these fibre groups was astonishingly high; in fact, according to the values of *d*_MK_, all (five) investigated CFs were the most matured, followed by the majority of PFs, whereas the majority of AFs were the least matured. The results of this work underline the potential of *d*_MK_ as a useful parameter for the study of brain development and largely strengthen our previous findings [57]. 

The ages of peak/minimum are important indices used in DTI studies to assess the heterochronicity of maturation and decline in different anatomic regions. That is, the higher the age of peak/minimum is, the more protractive maturation is. In this context, a new finding of the present work refers to the observation of strikingly strong correlations between *d*_MK_ (our work) and peaks/minima ages for FA/MD (published in an independent study [22]). However, it should be noted that the estimation of peak/minimum ages requires very large sample sizes distributed across a very broad age interval, i.e., a life span, in order to enable non-linear fits of age-dependent evolution trajectories with reasonable statistical accuracy. This is due to the relatively high inter-subject variability of diffusion MRI metrics, which, in cross-sectional studies, can mask real age-related changes for an individual. In turn, the fitted values of ages of peak/minimum exhibit rather large standard deviations, such as ca. 6–10 years for the FA peak, reported by Kochunov et al. [21], and 8–30 years for the RD minimum, reported by Hasan et al. [92]. Moreover, the evaluated ages of the peak/minimum depend on the selected fitting functions [16], such as quadratic [21,92], exponential [8,95] or Poisson [22], and can also be influenced by fitting algorithms. These, and other factors, may lead to large differences between the estimations, such as, for example, 27.6 years [22] versus 38.9 ± 6.6 years [21] reported for the FA peak in the fronto-occipital fasciculi in different works or 44.6 years [66] versus 35.0 years [22] reported for the FA peak of the CST. In addition, the ages of peak/minima cannot be estimated for fibres that support linear rather than non-linear dependence in the investigated age interval [64,66]. In view of all of these confounding factors, our results suggest that KT metrics, and in particular the *d*_MK_ of differently aged groups, may become robust biomarkers for the elucidation of heterochronous maturation in moderately sized subject samples, thereby potentially avoiding the need for the full time-consuming measurements of lifespan age dependences. In turn, *d*_MK_ in small subject samples may be a helpful test parameter at the pre-measurement planning stage of any comprehensively large statistical study.

### 4.4. Evidence of the Heterochronicity of Fibre Maturation Based on d_MK_

Tendentially, late-peaking tracts are mostly represented by AFs, such as the cingulum, UF and SLF. The latter are frontal-temporal connections that were shown to mature slowly during childhood and adolescence [10,22] and exhibit prolonged development and late decline. Thus far, the latest age trend reversal has been reported for the cingulum [21,22], with the peak/minimum at around 40 years old. Also, in our work, the Cg and Ch showed the largest *d*_MK_ (4.07 and 3.97, respectively) among all of the fibres studied. The cingulum is a complex WM tract constituting an important part of the limbic system and is associated with executive function, decision-making, and emotion. It is located beneath the cingulate cortex and contains connections entering and exiting the cingulate gyrus, as well as projections between prefrontal and parahippocampal cortices [96,97]. Therefore, late maturation of the cingulum bundle is assumed to underlie the protracted development of emotional and cognitive processes supported by these connections. In particular, a comparison of structure–function relationships in children and adults has shown that posterior cingulate–medial prefrontal cortex connectivity along the cingulum bundle is the most immature link in the default mode network of children [98]. In addition, several other fibres with late trend reversals, such as the UF, SLF, and SFOF [21,22,92], exhibited very large values of *d*_MK_. In particular, the long-lasting maturation of the SLF, a large AF bundle—connecting the cortical regions of the frontal, parietal, temporal, and occipital lobes—is associated with playing an important role in emotional regulation, executive functioning, and language processing [99,100]. The UF is another prominent long-range tract connecting the orbito-frontal cortex and the Brodmann area 10 with the anterior temporal lobes. Although its exact function is not well understood, it has been suggested that it plays an important role in episodic memory, language processing, and social/emotional functioning [101,102]. As a part of the frontotemporal network, it is also associated with several age-related and psychiatric disorders [101,102], including schizophrenia [103,104], primary progressive aphasia [105], temporal lobe epilepsy [106], and impaired error monitoring in a visual object–location association task [107]. More generally, it has been shown [108] that late-maturing WM tracts are likely to appear more sensitive to the pathophysiology of schizophrenia and are more susceptible to a faster age-related decline in FA values. Thus, studies and biomarkers of anachronous regional development might be of paramount importance for clinical diagnostics and for deepening our understanding of developmental pathologies. 

The *d*_MK_ values found in the group of CFs (FCB, TAP, and major partitions of the corpus callosum), crucial for inter-hemispheric interactions, were the lowest among those studied, indicating that these fibres were the most mature in our group of children. In particular, the fornix represents an example of the earliest peaking fibres with the ages of peak/minimum equal to 19.5/17.8 years [22] and, correspondingly, the *d*_MK_ value close to zero. Also, the Cohen’s *d* values of DT parameters in partitions of the corpus callosum were all very small (~0.1), which is in agreement with other DTI studies [8,58,109] showing an early rise of FA that already reaches 90% of its maximum by 11 years of age. The early development of these fibres can be associated with their basic functions, such as integrating information, visual perception, and language [110,111,112]. 

A unique aspect of our findings is that, in contrast to the Cohen’s *d* of DT parameters in the corpus callosum, which are small on an absolute scale (<0.2), the values of *d*_MK_ and the Cohen’s *d* of other KT parameters, although relatively small in comparison to other fibres, were medium to large on the absolute scale (*d*_MK_ = 0.28, 1.08, and 1.46 in the BCC, GCC, and SCC, respectively). This finding furnishes evidence of ongoing microstructural development in the corpus callosum beyond pre-adolescence (although not captured by DT metrics) and may provide further inferences in the biophysics of developmental microstructural changes. 

A further valuable finding of this study is the observation of characteristic patterns in the profiles of between-group differences. These patterns were mostly consistent for the C-P and A-P directions and more reliable in KT metrics compared to DT metrics. Our observations strongly support the existence of gradients in the timing of WM maturation consistent with those predicted in the literature. 

### 4.5. Underlying Neurobiological Aspects

Neurobiological mechanisms of diffusion non-Gaussianity in brain tissue giving rise to positive kurtosis values are multiple and are not yet fully understood. In development, they are usually associated with myelination, axonal growth, and changes in axonal packing [8,113,114,115]. Our work has shown that the strong increase in diffusional kurtosis from childhood to middle adulthood is widespread across WM and has different magnitudes for different fibres. This increase indicates a protracted formation of diffusional barriers and may provide additional insight into the mechanisms of microstructural reorganisation during late maturation. 

The common understanding of microstructural tissue changes in early childhood suggests that these changes are predominately due to myelination and axonal reorganisation, which is particularly rapid in the first two years of life. During this rapid change, the same mechanisms (myelination and axonal reorganisation) should dominate the evolution of both the DT and DK metrics: rapid increase in WM anisotropy, a decrease in MD, and an increase in diffusional kurtosis. However, some differences between the age trajectories of FA and MK values reported by Paydar et al. [58] suggest that additional mechanisms may also contribute to the evolution of MK during this early period. Thereafter, the developmental processes continue at a generally slower rate until adulthood. However, as this and our previous work [57] show, the mechanisms of microstructural reorganisation between pre-adolescence and adulthood are such that they cause greater diffusional kurtosis changes than changes in diffusivity and fractional anisotropy. 

Considering multi-compartment models of WM [56,116,117,118], with the simplest realisation being two water pools linked to the intra-axonal and extra-axonal spaces, larger diffusional kurtosis can result from increasing axonal density (i.e., an increase in the water fraction in the intra-axonal space) or decreasing water diffusivity in the extra-axonal space. Due to the limited range of *b*-factors (≤2.8 ms/µm^2^) and strongly restricted diffusion in the intra-axonal space, the slope of the slow diffusion component (in the plot of the signal vs. *b*-factors) cannot be accurately resolved. Thus, a high sensitivity of diffusion kurtosis measures to the changes in (apparent) water diffusivity in the extra-axonal space is unlikely. Consequently, we hypothesise that our results provide strong evidence of ongoing increasing axonal density/volume with age after pre-adolescence. This consideration can explain the differences in the sensitivity of diffusivity and diffusion kurtosis measures to late development tissue reorganisation. Kurtosis differences for various fibres can, in turn, be attributed to the corresponding differences in axonal water fractions and in the rates of their increase with age. Interestingly, kurtosis changes in the axial direction are not much smaller than those in the radial direction—compare *d*_AK_ and *d*_RK_ in Table 3. This indicates that significant fibre reorganisation also takes place in the axial direction. In particular, the formation or remodelling of crossing fibres and increasing neurite orientation dispersion, as described by the NODDI model [119], may contribute to changes in radial and axial diffusion kurtoses with age. 

The ability to detect microstructural variations in the neural substrates non-invasively allows inferences on both normal and abnormal cognitive development to be made, and provides a valuable tool for the assessment of various treatment strategies. The high sensitivity of DK parameters to subtle microstructural remodelling beyond pre-adolescence makes them potentially attractive covariates for the study of cognitive development in combined studies of functional and structural connectivity. In particular, the use of *d*_MK_ can be expected to be a promising, robust parameter in future studies. Firstly, the large age-related effect size associated with this parameter can be beneficial for longitudinal studies (with fixed intervals between the starting and final ages), in which DT metrics can be expected to provide only incremental changes [109]. Secondly, it can be used in studies with only moderate sample sizes available for between-group comparisons, such as studies aiming to compare neuro-psychological scores and functional capabilities in differently aged groups. 

The rationale for choosing DKI over other biophysical models to analyse the diffusion MRI signal (such as NODDI [119]) relates to the fact that DKI makes no assumptions about the underlying tissue microstructure. To a greater or lesser extent, the majority of the biophysical models available in the literature make assumptions regarding the tissue microstructure or diffusion properties and, therefore, any deviation of the sample group from a specific assumption of a biophysical model may lead to bias in the corresponding model parameters, hence losing the desired specificity [120]. 

### 4.6. Limitations of the Study

The main limitation of this work predominately relates to the relatively small group size and the absence of longitudinal measurements. On the other hand, very large effect size values observed with *d*_MK_ in various group comparisons allow one to ameliorate this limitation. Nevertheless, a more comprehensive study should benefit from being longitudinal in design and from including a greater number of subjects across a broader age interval from childhood to adulthood. Some other technical limitations, such as the influence of motion, eddy-currents, co-registration approach, etc., have been discussed in detail in a previous study [57]. A potentially important confounding factor in the present work is the partial volume effect between different fibre bundles, or between tissue and CSF, which is particularly prominent in diffusion MRI experiments performed under conventional resolution settings, i.e., with a voxel size in the order of 8 mm^3^. In this regard, more sophisticated models able to account for this effect, such as the DTI or DKI with free water elimination [121,122,123,124], may prove to be more specific and robust in these types of analyses than their conventional counterparts.

## 5. Conclusions

We observed a unique sensitivity of DKI metrics as biomarkers of microstructural development and regional heterochronicity of various anatomic regions between pre-adolescence and middle adulthood. The increase in diffusional kurtosis after pre-adolescence indicates a protracted formation of diffusional barriers and, hence, can provide additional insights into the mechanisms of microstructural reorganisation during late maturation. Developmental changes were observed across the majority of anatomic regions and were substantially better captured by KT compared to DT metrics. In particular, Cohen’s *d* of the mean kurtosis revealed itself as a promising parameter for ranking the maturity of various fibres and, beyond that, different fibre types as well (AFs, PFs, and CFs). Based on this parameter, most fibres that exhibit more protracted maturation belong to AFs, whereas the most matured fibres belong to CFs. At the same time, relatively elevated values of *d*_MK_ suggest that the maturation of CFs is still ongoing in these fibres, although at slower rates compared to other fibre types. 

Our data provide important evidence for the existence of gradients in the timing of WM maturation. Here, we refer to striking differences in the profile shapes of *d*_MK_ for R-L, P-A, and I-S directions, which tend to be in accordance with those predicted in the literature. Beyond that, a strong correlation of *d*_MK_ with the age of peak for FA and MD suggests that *d*_MK_, in relatively small groups—due to its proven large effect size in the context of maturation processes—might serve as a promising preliminary test parameter when planning comprehensive studies with thousands of participants involved to ensure the statistical significance of the results. 

It can be assumed that the biophysical mechanism underlying the different sensitivities of DT and KT metrics to developmental tissue remodelling is predominantly due to the greater effect of increasing axonal density/volume on KT than on DT indices. In turn, the regional heterogeneity of KT metrics and heterochronicity in the maturation of various fibres can also be, at least partially, attributed to the corresponding differences in axonal density/volume and the rates at which axonal water fractions increase with age in different fibres. Large effect sizes were found in between-group comparisons with Cohen’s *d* values of KT metrics. More generally, the high sensitivity of DKI parameters to subtle spatiotemporal microstructural reorganisation beyond pre-adolescence makes them also potentially attractive covariates for the study of cognitive development.

## Figures and Tables

**Table 1:** Abbreviations of WM tracts provided by the Johns Hopkins University (JHU) Atlas “ICBM-DTI-81” investigated in this work.

Number	WM Tract	Abbreviation
1.	Middle cerebellar peduncle	MCP
2.	Pontine crossing tract	PCT
3.	Genu of corpus callosum	GCC
4.	Body of corpus callosum	BCC
5.	Splenium of corpus callosum	SCC
6.	Fornix column and body	FCB
7.	Corticospinal tract	CST
8.	Medial lemniscus	ML
9.	Inferior cerebellar peduncle	ICP
10.	Superior cerebellar peduncle	SCP
11.	Cerebral peduncle	CP
12.	Anterior limb of internal capsule	ALIC
13.	Posterior limb of internal capsule	PLIC
14.	Retrolenticular part of internal capsule	RPIC
15.	Anterior corona radiata	ACR
16.	Superior corona radiata	SCR
17.	Posterior corona radiata	PCR
18.	Posterior thalamic radiation (including optic radiation)	PTR
19.	Sagittal stratum (including inferior longitudinal fasciculus and inferior fronto-occipital fasciculus)	SS
20.	External capsule	EC
21.	Cingulum (cingulate gyrus)	Cg
22.	Cingulum (hippocampus)	Ch
23.	Fornix (crus) stria terminalis	FST
24.	Superior longitudinal fasciculus	SLF
25.	Superior fronto-occipital fasciculus	SFOF
26.	Uncinate fasciculus	UF
27.	Tapetum	TAP

**Table 2:** Between-group differences in percentage, absolute values of Cohen’s *d*, and values of *p* for DT parameters in WM ROIs. The values for all fibres were evaluated by averaging the absolute values of percentual changes and Cohen’s *d* across all fibres. Significant between-group differences are indicated by asterisks based on the two-sided Student’s *t*-test analysis (*p* ≤ 0.00185, Bonferroni corrected).

	FA			MD			AD			RD		
WM Tract	Δ [%]	*d*_FA_	*p*	Δ [%]	*d*_MD_	*p*	Δ [%]	*d*_AD_	*p*	Δ [%]	*d*_RD_	*p*
MCP	0.5	0.17	6 × 10^−1^	−3.7	1.07	2 × 10^−3^	−4.8	1.45	6 × 10^−5^ *	−2.6	0.64	5 × 10^−2^
PCT	0.0	0.00	1 × 10^0^	−10	1.55	2 × 10^−5^ *	−10.3	1.95	4 × 10^−7^ *	−9.7	1.17	8 × 10^−4^ *
GCC	−0.5	0.12	7 × 10^−1^	0.8	0.12	7 × 10^−1^	−0.1	0.02	1 × 10^0^	1.8	0.2	5 × 10^−1^
BCC	2.8	0.51	1 × 10^−1^	0.5	0.1	8 × 10^−1^	1.6	0.45	2 × 10^−1^	−0.8	0.08	8 × 10^−1^
SCC	1.1	0.36	3 × 10^−1^	0.4	0.1	8 × 10^−1^	0.4	0.11	7 × 10^−1^	0.6	0.09	8 × 10^−1^
FCB	−6.4	0.57	8 × 10^−2^	13.5	1.13	1 × 10^−3^ *	11	1.33	2 × 10^−4^ *	15.8	1.00	3 × 10^−3^
CST	5.5	1.18	7 × 10^−4^ *	−2.0	0.4	2 × 10^−1^	0.1	0.01	1 × 10^−1^	−4.2	0.61	6 × 10^−2^
ML	1.3	0.32	3 × 10^−1^	−2.3	0.49	1 × 10^−1^	−1.5	0.31	3 × 10^−1^	−3.2	0.57	8 × 10^−2^
ICP	7.8	1.24	4 × 10^−4^ *	−0.2	0.07	8 × 10^−1^	1.9	0.72	3 × 10^−2^	−2.3	0.44	8 × 10^−1^
SCP	5.1	1.20	6 × 10^−4^ *	−2.6	0.47	2 × 10^−1^	−0.2	0.05	9 × 10^−1^	−5.2	0.66	5 × 10^−2^
CP	−3.0	0.73	3 × 10^−2^	0.7	0.16	6 × 10^−1^	−2.1	0.64	6 × 10^−2^	4.5	0.58	8 × 10^−2^
ALIC	4.5	1.11	1 × 10^−3^ *	0.4	0.08	8 × 10^−1^	1.1	0.26	4 × 10^−1^	−0.4	0.05	9 × 10^−1^
PLIC	−1.0	0.34	3 × 10^−1^	−4.2	1.12	1 × 10^−3^ *	−5.7	1.80	2 × 10^−6^ *	−1.7	0.28	4 × 10^−1^
RPIC	−3.2	0.87	1 × 10^−2^	−1.3	0.34	3 × 10^−1^	−4.1	1.10	1 × 10^−3^ *	2.4	0.42	2 × 10^−1^
ACR	−1.6	0.30	4 × 10^−1^	−2.5	0.53	1 × 10^−1^	−3.6	0.99	4 × 10^−3^	−1.3	0.21	5 × 10^−1^
SCR	−2.9	0.67	4 × 10^−2^	−1.5	0.32	3 × 10^−1^	−3.6	0.82	1 × 10^−2^	0.8	0.14	7 × 10^−1^
PSR	−4.1	0.84	1 × 10^−2^	−1.8	0.37	2 × 10^−1^	−4.3	1.06	2 × 10^−3^	0.9	0.14	7 × 10^−1^
PTR	−2.4	0.57	8 × 10^−2^	−3.4	0.8	2 × 10^−2^	−4.9	1.29	2 × 10^−4^ *	−1.6	0.28	4 × 10^−1^
SS	0.6	0.12	7 × 10^−1^	−2.6	0.55	1 × 10^−1^	−2.2	0.53	1 × 10^−1^	−3.1	0.46	2 × 10^−1^
EC	0.6	0.14	7 × 10^−1^	6.7	1.44	7 × 10^−5^ *	6.1	1.64	9 × 10^−6^ *	7.3	1.22	5 × 10^−4^ *
Cg	5.3	0.96	5 × 10^−3^	2.0	0.56	9 × 10^−2^	3.8	0.87	9 × 10^−3^	0.3	0.07	8 × 10^−1^
Ch	6.4	1.07	2 × 10^−3^ *	−3.0	0.94	6 × 10^−3^	−2.6	0.75	2 × 10^−2^	−3.4	0.86	1 × 10^−2^
FST	−3.2	0.58	8 × 10^−2^	−2.6	0.5	1 × 10^−1^	−5	1.21	6 × 10^−4^ *	0.0	0.00	1 × 10^0^
SLF	1.3	0.31	3 × 10^−1^	−0.1	0.02	9 × 10^−1^	0.1	0.03	9 × 10^−1^	−0.3	0.06	9 × 10^−1^
SFOF	0.4	0.06	8 × 10^−1^	1.0	0.12	7 × 10^−1^	0.2	0.03	9 × 10^−1^	1.8	0.18	6 × 10^−1^
UF	4.9	0.88	9 × 10^−3^	−2.5	0.73	3 × 10^−2^	−1.5	0.42	2 × 10^−1^	−3.5	0.79	2 × 10^−2^
TAP	3.2	0.49	1 × 10^−1^	−5.5	0.64	5 × 10^−2^	−4.7	0.63	6 × 10^−2^	−6.2	0.63	6 × 10^−2^
All fibres	2.9	0.58		2.9	0.55		3.2	0.75		3.1	0.43	

**Table 3:** Between-group differences in percentage, absolute values of Cohen’s *d*, and values of *p* for KT parameters in WM ROIs. The values for all fibres were evaluated by averaging the absolute values of percentual changes and Cohen’s *d* across all fibres. Significant between-group differences are indicated by asterisks based on the two-sided Student’s *t*-test analysis (*p* ≤ 0.00185, Bonferroni corrected).

	KA			MK			AK			RK		
WM Tract	Δ [%]	*d*_KA_	*p*	Δ [%]	*d*_MK_	*p*	Δ [%]	*d*_AK_	*p*	Δ [%]	*d*_RK_	*p*
MCP	16.8	2.36	6 × 10^−9^ *	10	2.35	8 × 10^−9^ *	6.1	1.41	9 × 10^−5^ *	13.8	2.64	4 × 10^−10^ *
PCT	20.3	1.87	9 × 10^−7^ *	16.1	3.19	3 × 10^−12^ *	10.2	2.26	2 × 10^−8^ *	16.3	2.23	2 × 10^−8^ *
GCC	2.3	0.24	5 × 10^−1^	5.4	1.08	2 × 10^−3^ *	10.1	2.38	6 × 10^−9^ *	7.9	1.08	2 × 10^−3^ *
BCC	7.4	0.77	2 × 10^−2^	1.5	0.28	4 × 10^−1^	8.9	2	3 × 10^−7^ *	1.4	0.17	6 × 10^−1^
SCC	16.6	1.76	3 × 10^−6 *^	7	1.46	5 × 10^−5^ *	8.1	1.62	1 × 10^−5^ *	11.6	1.66	7 × 10^−6^ *
FCB	−12.5	0.62	6 × 10^−2^	0.1	0.01	1 × 10^0^	−1.3	0.26	4 × 10^−1^	−1.6	0.15	7 × 10^−1^
CST	17.3	2.15	5 × 10^−8^ *	9.7	2.07	1 × 10^−7^ *	3.4	0.76	2 × 10^−2^	16.6	2.57	9 × 10^−10^ *
ML	8.6	0.97	4 × 10^−3^	11	2.01	2 × 10^−7^ *	6.6	1.28	3 × 10^−4^ *	11.8	1.62	1 × 10^−6^ *
ICP	22.4	2.51	2 × 10^−9^ *	11.4	2.72	2 × 10^−10^ *	4.4	1.09	2 × 10^−3^ *	15.8	2.58	9 × 10^−10^ *
SCP	21.6	2.21	3 × 10^−8^ *	7.8	1.58	2 × 10^−5^ *	3.9	0.85	1 × 10^−2^	12.9	2.31	1 × 10^−8^ *
CP	12.6	1.27	3 × 10^−4^ *	11.1	2.15	5 × 10^−8^ *	13	2.35	9 × 10^−9^ *	17.3	2.4	4 × 10^−8^ *
ALIC	12.9	1.47	5 × 10^−5^ *	11.7	2.43	4 × 10^−9^ *	7.8	1.98	3 × 10^−7^ *	11.1	1.86	1 × 10^−6^ *
PLIC	9.5	1.22	5 × 10^−4^ *	10	1.87	9 × 10^−7^ *	14.3	2.8	1 × 10^−10^ *	13.2	1.74	3 × 10^−6^ *
RPIC	5.3	0.68	4 × 10^−2^	10.9	2.3	1 × 10^−8^ *	14.6	3.12	6 × 10^−12^ *	11.5	1.67	6 × 10^−6^ *
ACR	4.7	0.43	2 × 10^−1^	9	1.88	8 × 10^−7^ *	8.6	2.33	9 × 10^−9^ *	10.8	1.78	2 × 10^−6^ *
SCR	7	0.78	2 × 10^−2^	8.7	2.01	2 × 10^−7^ *	10.3	2.6	8 × 10^−10^ *	7.2	1.3	2 × 10^−4^ *
PSR	10.6	1.05	2 × 10^−3^	10.5	2.02	2 × 10^−7^ *	9.7	1.96	4 × 10^−7^ *	10.8	1.64	9 × 10^−6^ *
PTR	7	0.78	2 × 10^−2^	10.7	1.98	3 × 10^−7^ *	11.5	2.41	5 × 10^−9^ *	12	1.58	2 × 10^−5^ *
SS	1.7	0.18	6 × 10^−1^	12.9	2.32	1 × 10^−8^ *	12.4	2.38	7 × 10^−9^ *	14	1.9	7 × 10^−7^ *
EC	3.2	0.38	2 × 10^−1^	19.9	3.83	1 × 10^−14^ *	11.7	3.31	1 × 10^−12^ *	25.6	3.47	3 × 10^−13^ *
Cg	16.5	2.25	2 × 10^−8^ *	18.4	4.07	2 × 10^−15^ *	7.9	1.79	2 × 10^−6^ *	23.5	3.1	7 × 10^−12^ *
Ch	27.5	2.9	4 × 10^−11^ *	32.7	3.97	5 × 10^−15^ *	19.8	3.33	1 × 10^−12^ *	36.2	3.83	7 × 10^−14^ *
FST	10.8	1.1	2 × 10^−3^ *	13.9	2.48	2 × 10^−9^ *	17.3	3.19	4 × 10^−12^ *	10	1.28	3 × 10^−4^ *
SLF	6.1	0.76	2 × 10^−2^	10.4	2.7	3 × 10^−10^ *	8.8	2.15	6 × 10^−8^ *	12.4	2.33	1 × 10^−8^ *
SFOF	17	1.3	2 × 10^−4^ *	13.9	2.39	5 × 10^−9^ *	8.8	1.84	1 × 10^−6^ *	18	2.22	3 × 10^−8^ *
UF	15.4	1.57	2 × 10^−5^ *	18.7	3.04	1 × 10^−11^ *	16.8	2.9	4 × 10^−11^ *	19.2	1.93	5 × 10^−7^ *
TAP	17.5	1.11	1 × 10^−3^ *	3	0.29	4 × 10^−1^	2.1	0.35	3 × 10^−1^	6.9	0.43	2 × 10^−1^
All fibres	12.3	1.28		11.3	2.17		9.6	2.02		13.7	1.90	

**Table 4:** Pearson correlation coefficients for pairs of Cohen’s *d* values of various DT and KT parameters. Bold numbers denote large (≥0.5), significant (*, *p* < 0.05) correlations.

	*d*_MD_	*d*_AD_	*d*_RD_	*d*_FA_	*d*_MK_	*d*_AK_	*d*_RK_	*d*_KA_
*d*_MD_	1	**0.89** *	**0.85** *	0.00	−0.08	−0.17	−0.06	−0.41 *
*d*_AD_		1	**0.52** *	0.42 *	−0.04	−0.34	0.03	−0.15
*d*_RD_			1	**−0.50** *	−0.14	0.09	−0.17	**−0.60** *
*d*_FA_				1	0.25	−0.29	0.40 *	**0.64** *
*d*_MK_					1	**0.56** *	**0.88** *	0.49 *
*d*_AK_						1	0.36	−0.04
*d*_RK_							1	**0.63** *
*d*_KA_								1

**Table 5:** The values of R^2^, F-stat, and *p*-values for the fits of the data points in Figure 5a,b using linear regression models (see Section 2.3).

	MD	AD	RD	FA	MK	AK	RK	KA
**R-L (left)**								
R^2^ (linear)	0.18	0.25	0.12	0.44	0.81	0.61	0.68	0.003
F-stat (linear)	14	20	9	48	270	97	131	0.2
*p*-value (linear)	<10^−3^	<10^5^	4.6 × 10^−3^	<10^−5^	<10^−20^	<10^−10^	<10^−10^	6.9 × 10^−1^
R^2^ (quadratic)	0.92	0.90	0.86	0.44	0.84	0.72	0.68	0.06
F-stat (quadratic)	348	264	189	24	159	78	65	2
*p*-value (quadratic)	<10^−20^	<10^−20^	<10^−20^	<10^−5^	<10^−20^	<10^−10^	<10^−10^	1.4 × 10^−1^
**R-L (right)**								
R^2^ (linear)	0.09	0.14	0.06	0.36	0.86	0.69	0.72	0.09
F-stat (linear)	6	10	4	34	366	133	156	6
*p*-value (linear)	1.8 × 10^−2^	2.5 × 10^−3^	5.7 × 10^−2^	<10^−5^	<10^−20^	<10^−10^	<10^−10^	2.1 × 10^−2^
R^2^ (quadratic)	0.45	0.54	0.34	0.36	0.86	0.69	0.73	0.35
F-stat (quadratic)	24	34	15	17	181	66	79	16
*p*-value (quadratic)	<10^−5^	<10^−5^	<10^−5^	<10^−5^	<10^−20^	<10^−10^	<10^−10^	<10^−5^
**R-L**								
R^2^ (quadratic)	0.31	0.39	0.24	0.37	0.82	0.67	0.65	0.09
F-stat (quadratic)	28	39	19	36	279	124	112	6
*p*-value (quadratic)	<10^−5^	<10^−10^	<10^−5^	<10^−10^	<10^−20^	<10^−20^	<10^−20^	3.5 × 10^−3^
**P-A**								
R^2^ (linear)	0.49	0.51	0.44	0.04	0.67	0.65	0.70	0.48
F-stat (linear)	151	168	125	6	324	294	379	150
*p*-value (linear)	<10^−20^	<10^−20^	<10^−20^	1.4 × 10^−2^	<10^−20^	<10^−20^	<10^−20^	<10^−20^
R^2^ (quadratic)	0.69	0.68	0.65	0.18	0.68	0.80	0.70	0.50
F-stat (quadratic)	177	172	150	17	169	325	190	80
*p*-value (quadratic)	<10^−20^	<10^−20^	<10^−20^	<10^−5^	<10^−20^	<10^−20^	<10^−20^	<10^−20^
**I-S**								
R^2^ (linear)	0.66	0.55	0.67	0.15	0.18	0.03	0.27	0.02
F-stat (linear)	239	152	257	22	27	4	46	2
*p*-value (linear)	<10^−20^	<10^−20^	<10^−20^	<10^−5^	<10^−5^	4.4 × 10^−2^	<10^−5^	1.2 × 10^−1^
R^2^ (quadratic)	0.77	0.79	0.70	0.56	0.21	0.61	0.27	0.48
F-stat (quadratic)	205	233	146	79	16	96	23	56
*p*-value (quadratic)	<10^−20^	<10^−20^	<10^−20^	<10^−20^	<10^−5^	<10^−20^	<10^−5^	<10^−10^

## Data Availability

The datasets generated and/or analysed during the current study are subject to some legal and ethical restrictions, which are also stated in the informed consent form approved by the Ethics Committee of the Medical Faculty of RWTH Aachen University and signed by each participant. The general rules of the Forschungszentrum Juelich, RWTH Aachen University and the Ethics Committee of the Medical Faculty of RWTH Aachen University provide the following procedure for the sharing of anonymised MRI data: Data may be shared with interested researchers whose identity and affiliation to a research institution must be ensured and confirmed. It must be made clear that the data will only be used by the interested researchers for research purposes in accordance with the provisions of the Declaration of Helsinki and in accordance with the provisions of the applicable data protection regulations (The General Data Protection Regulation). All data used for this publication are available upon request via the following institutional point of contact who can field data inquiries from fellow researchers: Secretary’s office of the Institute of Neuroscience and Medicine (INM-4) Forschungszentrum Jülich Secretary: Nancy Malsbenden building: 15.14, room: 201 phone: +49-2461-61-85575 fax: +49-2461-61-1919 INM4-Sekretariat@fz-juelich.de.

## Supplementary Materials

The following supporting information can be downloaded at: https://www.mdpi.com/article/10.3390/brainsci14050495/s1. Table S1: Mean and standard deviation values of DT parameters in various WM ROIs; Table S2: Mean and standard deviation values of KT parameters in various WM ROIs.

## Abbreviations

GM	grey matter
WM	white matter
DTI	diffusion tensor imaging
MD	mean diffusivity
FA	fractional anisotropy
AF	association fibre
CF	commissural fibre
PF	projection fibre
P-A	posterior-to-anterior
C-P	central-to-peripheral
I-S	inferior-to-superior
DKI	diffusion kurtosis imaging
MK	mean kurtosis
KT	kurtosis tensor
AD	axial diffusivity
RD	radial diffusivity
AK	axial kurtosis
RK	radial kurtosis
KA	kurtosis anisotropy
R-L	right-to-left
