# Supplementary material for: Spatiotemporal Patterns of White Matter Maturation after Pre-Adolescence: A Diffusion Kurtosis Imaging Study
Source: Brain Sci. 2024 May 13;14(5):495. doi: 10.3390/brainsci14050495 (PMC11119177; doi:10.3390/brainsci14050495)
Supplement: Supplementary file 1 [file brainsci-14-00495-s001.zip › brainsci-2984481-supplementary.pdf]

Table S1. Mean values (M) and standard deviations (SD) of DT parameters in various WM ROIs for adults and children.

	FA		MD [μm²/ms]		AD [μm²/ms]		RD [μm²/ms]	
	**adult**	**child**	**adult**	**child**	**adult**	**child**	**adult**	**child**
WM tract	M ± SD	M ± SD	M ± SD	M ± SD	M ± SD	M ± SD	M ± SD	M ± SD
MCP	0.45 ± 0.01	0.45 ± 0.01	0.94 ± 0.03	0.97 ± 0.03	1.39 ± 0.05	1.46 ± 0.04	0.71 ± 0.03	0.73 ± 0.03
PCT	0.42 ± 0.02	0.42 ± 0.02	0.82 ± 0.06	0.91 ± 0.06	1.18 ± 0.07	1.32 ± 0.07	0.64 ± 0.06	0.70 ± 0.06
GCC	0.54 ± 0.03	0.54 ± 0.02	1.06 ± 0.07	1.05 ± 0.06	1.76 ± 0.08	1.76 ± 0.09	0.70 ± 0.07	0.69 ± 0.05
BCC	0.55 ± 0.03	0.53 ± 0.03	1.16 ± 0.06	1.16 ± 0.07	1.93 ± 0.07	1.90 ± 0.07	0.78 ± 0.07	0.78 ± 0.07
SCC	0.59 ± 0.02	0.59 ± 0.02	1.09 ± 0.06	1.09 ± 0.04	1.88 ± 0.08	1.88 ± 0.05	0.70 ± 0.05	0.69 ± 0.04
FCB	0.38 ± 0.04	0.40 ± 0.05	2.15 ± 0.25	1.89 ± 0.19	3.00 ± 0.25	2.71 ± 0.19	1.72 ± 0.27	1.48 ± 0.20
CST	0.50 ± 0.02	0.48 ± 0.02	0.94 ± 0.05	0.96 ± 0.04	1.47 ± 0.07	1.46 ± 0.06	0.67 ± 0.05	0.70 ± 0.04
ML	0.53 ± 0.02	0.52 ± 0.02	0.88 ± 0.05	0.90 ± 0.03	1.47 ± 0.10	1.49 ± 0.04	0.59 ± 0.04	0.61 ± 0.03
ICP	0.44 ± 0.02	0.41 ± 0.03	0.96 ± 0.03	0.97 ± 0.03	1.45 ± 0.04	1.42 ± 0.03	0.72 ± 0.04	0.74 ± 0.04
SCP	0.55 ± 0.02	0.52 ± 0.02	1.33 ± 0.08	1.36 ± 0.07	2.15 ± 0.10	2.15 ± 0.08	0.92 ± 0.08	0.97 ± 0.07
CP	0.57 ± 0.02	0.59 ± 0.02	1.02 ± 0.06	1.01 ± 0.03	1.73 ± 0.06	1.77 ± 0.05	0.66 ± 0.06	0.64 ± 0.04
ALIC	0.52 ± 0.02	0.49 ± 0.02	0.84 ± 0.06	0.83 ± 0.03	1.36 ± 0.07	1.35 ± 0.04	0.57 ± 0.05	0.58 ± 0.02
PLIC	0.63 ± 0.02	0.64 ± 0.02	0.78 ± 0.03	0.82 ± 0.03	1.43 ± 0.04	1.52 ± 0.06	0.46 ± 0.03	0.46 ± 0.03
RPIC	0.55 ± 0.02	0.57 ± 0.02	0.91 ± 0.03	0.92 ± 0.04	1.52 ± 0.05	1.58 ± 0.06	0.61 ± 0.04	0.59 ± 0.04
ACR	0.43 ± 0.03	0.44 ± 0.02	0.86 ± 0.05	0.88 ± 0.03	1.28 ± 0.05	1.33 ± 0.04	0.65 ± 0.05	0.66 ± 0.03
SCR	0.48 ± 0.02	0.49 ± 0.02	0.82 ± 0.04	0.83 ± 0.04	1.27 ± 0.06	1.32 ± 0.06	0.59 ± 0.04	0.59 ± 0.04
PSR	0.44 ± 0.02	0.46 ± 0.02	0.92 ± 0.05	0.94 ± 0.04	1.39 ± 0.07	1.45 ± 0.05	0.69 ± 0.05	0.68 ± 0.04
PTR	0.50 ± 0.02	0.52 ± 0.02	0.96 ± 0.04	1.00 ± 0.04	1.55 ± 0.06	1.63 ± 0.06	0.67 ± 0.04	0.68 ± 0.04
SS	0.49 ± 0.03	0.49 ± 0.02	0.97 ± 0.06	1.00 ± 0.04	1.53 ± 0.08	1.56 ± 0.05	0.69 ± 0.06	0.71 ± 0.04
EC	0.40 ± 0.02	0.40 ± 0.01	0.94 ± 0.05	0.89 ± 0.02	1.37 ± 0.06	1.29 ± 0.03	0.73 ± 0.05	0.68 ± 0.02
Cg	0.39 ± 0.02	0.37 ± 0.02	0.93 ± 0.03	0.92 ± 0.03	1.36 ± 0.04	1.31 ± 0.07	0.72 ± 0.03	0.72 ± 0.03
Ch	0.37 ± 0.02	0.35 ± 0.02	0.91 ± 0.03	0.94 ± 0.03	1.27 ± 0.05	1.31 ± 0.04	0.72 ± 0.02	0.75 ± 0.04
FST	0.46 ± 0.03	0.47 ± 0.02	1.05 ± 0.06	1.08 ± 0.05	1.59 ± 0.07	1.67 ± 0.07	0.79 ± 0.07	0.79 ± 0.05
SLF	0.44 ± 0.02	0.43 ± 0.02	0.87 ± 0.04	0.87 ± 0.03	1.29 ± 0.04	1.29 ± 0.04	0.66 ± 0.04	0.66 ± 0.03
SFOF	0.47 ± 0.03	0.47 ± 0.03	0.81 ± 0.07	0.81 ± 0.06	1.26 ± 0.09	1.26 ± 0.08	0.59 ± 0.06	0.58 ± 0.05
UF	0.41 ± 0.02	0.39 ± 0.02	0.90 ± 0.04	0.92 ± 0.02	1.32 ± 0.05	1.34 ± 0.04	0.69 ± 0.04	0.71 ± 0.03
Tapetum	0.43 ± 0.02	0.41 ± 0.03	1.61 ± 0.16	1.71 ± 0.14	2.30 ± 0.20	2.41 ± 0.16	1.27 ± 0.14	1.35 ± 0.13

Table S2. Mean values (M) and standard deviations (SD) of KT parameters in various WM ROIs for adults and children.

	**KA**		**MK**		**AK**		**RK**	
	**adult**	**child**	**adult**	**child**	**adult**	**child**	**adult**	**child**
WM tract	M ± SD	M ± SD	M ± SD	M ± SD	M ± SD	M ± SD	M ± SD	M ± SD
MCP	0.44 ± 0.02	0.37 ± 0.03	1.15 ± 0.05	1.05 ± 0.04	0.77 ± 0.04	0.73 ± 0.02	1.46 ± 0.06	1.28 ± 0.07
PCT	0.42 ± 0.04	0.35 ± 0.03	1.25 ± 0.07	1.08 ± 0.04	0.90 ± 0.04	0.82 ± 0.03	1.62 ± 0.11	1.39 ± 0.09
GCC	0.42 ± 0.04	0.41 ± 0.04	0.97 ± 0.05	0.92 ± 0.04	0.63 ± 0.03	0.57 ± 0.02	1.34 ± 0.11	1.25 ± 0.07
BCC	0.42 ± 0.04	0.39 ± 0.04	0.93 ± 0.05	0.92 ± 0.05	0.61 ± 0.03	0.56 ± 0.02	1.27 ± 0.12	1.26 ± 0.08
SCC	0.49 ± 0.04	0.42 ± 0.04	1.04 ± 0.05	0.98 ± 0.04	0.58 ± 0.03	0.53 ± 0.02	1.47 ± 0.10	1.32 ± 0.09
FCB	0.14 ± 0.03	0.16 ± 0.04	0.64 ± 0.05	0.64 ± 0.05	0.50 ± 0.03	0.51 ± 0.02	0.79 ± 0.08	0.81 ± 0.09
CST	0.47 ± 0.03	0.40 ± 0.03	1.20 ± 0.06	1.09 ± 0.04	0.76 ± 0.04	0.74 ± 0.03	1.68 ± 0.10	1.44 ± 0.09
ML	0.43 ± 0.03	0.40 ± 0.04	1.12 ± 0.06	1.01 ± 0.05	0.73 ± 0.04	0.69 ± 0.03	1.56 ± 0.10	1.40 ± 0.11
ICP	0.35 ± 0.02	0.29 ± 0.03	1.05 ± 0.05	0.94 ± 0.03	0.78 ± 0.04	0.74 ± 0.02	1.29 ± 0.08	1.11 ± 0.06
SCP	0.40 ± 0.03	0.33 ± 0.03	1.00 ± 0.05	0.93 ± 0.04	0.58 ± 0.03	0.56 ± 0.02	1.36 ± 0.07	1.21 ± 0.07
CP	0.50 ± 0.04	0.45 ± 0.05	1.17 ± 0.06	1.05 ± 0.05	0.64 ± 0.04	0.57 ± 0.02	1.65 ± 0.10	1.41 ± 0.10
ALIC	0.46 ± 0.04	0.41 ± 0.03	1.05 ± 0.05	0.94 ± 0.04	0.69 ± 0.03	0.64 ± 0.01	1.37 ± 0.08	1.24 ± 0.07
PLIC	0.55 ± 0.03	0.50 ± 0.04	1.18 ± 0.06	1.07 ± 0.06	0.67 ± 0.03	0.58 ± 0.03	1.67 ± 0.11	1.48 ± 0.12
RPIC	0.46 ± 0.03	0.43 ± 0.04	1.11 ± 0.05	1.00 ± 0.05	0.75 ± 0.04	0.66 ± 0.02	1.42 ± 0.10	1.28 ± 0.08
ACR	0.36 ± 0.04	0.35 ± 0.03	1.02 ± 0.05	0.93 ± 0.04	0.80 ± 0.03	0.74 ± 0.02	1.20 ± 0.07	1.09 ± 0.06
SCR	0.41 ± 0.03	0.39 ± 0.04	1.11 ± 0.04	1.02 ± 0.05	0.82 ± 0.03	0.74 ± 0.02	1.38 ± 0.07	1.29 ± 0.08
PSR	0.37 ± 0.03	0.33 ± 0.03	1.01 ± 0.05	0.91 ± 0.04	0.78 ± 0.04	0.71 ± 0.03	1.23 ± 0.07	1.11 ± 0.07
PTR	0.41 ± 0.03	0.39 ± 0.04	0.97 ± 0.05	0.88 ± 0.04	0.72 ± 0.04	0.64 ± 0.02	1.21 ± 0.08	1.08 ± 0.08
SS	0.36 ± 0.03	0.35 ± 0.04	1.01 ± 0.06	0.89 ± 0.04	0.78 ± 0.05	0.70 ± 0.02	1.18 ± 0.08	1.04 ± 0.07
EC	0.31 ± 0.03	0.30 ± 0.02	0.91 ± 0.04	0.76 ± 0.04	0.76 ± 0.03	0.68 ± 0.02	1.04 ± 0.07	0.83 ± 0.06
Cg	0.35 ± 0.02	0.30 ± 0.02	0.95 ± 0.04	0.80 ± 0.03	0.78 ± 0.04	0.72 ± 0.02	1.12 ± 0.08	0.90 ± 0.06
Ch	0.33 ± 0.03	0.26 ± 0.02	0.98 ± 0.08	0.74 ± 0.04	0.82 ± 0.05	0.69 ± 0.03	1.10 ± 0.10	0.81 ± 0.05
FST	0.35 ± 0.03	0.32 ± 0.03	1.00 ± 0.06	0.88 ± 0.04	0.75 ± 0.05	0.64 ± 0.02	1.17 ± 0.10	1.07 ± 0.07
SLF	0.38 ± 0.03	0.36 ± 0.03	1.05 ± 0.04	0.95 ± 0.03	0.83 ± 0.04	0.77 ± 0.03	1.24 ± 0.06	1.10 ± 0.05
SFOF	0.47 ± 0.06	0.40 ± 0.05	1.09 ± 0.07	0.96 ± 0.04	0.74 ± 0.04	0.68 ± 0.02	1.40 ± 0.11	1.19 ± 0.08
UF	0.31 ± 0.03	0.27 ± 0.02	0.85 ± 0.05	0.72 ± 0.03	0.73 ± 0.04	0.63 ± 0.03	0.92 ± 0.09	0.77 ± 0.05
Tapetum	0.24 ± 0.03	0.20 ± 0.03	0.65 ± 0.08	0.63 ± 0.05	0.55 ± 0.04	0.54 ± 0.02	0.75 ± 0.13	0.70 ± 0.09
